# Population Coding and Correlated Variability in Electrosensory Pathways

**DOI:** 10.3389/fnint.2018.00056

**Published:** 2018-11-27

**Authors:** Volker Hofmann, Maurice J. Chacron

**Affiliations:** Department of Physiology, McGill University, Montréal, QC, Canada

**Keywords:** population coding, correlated variability, noise correlations, stimulus encoding, feedback, electric fish, correlation shaping, electrosensory lateral line lobe

## Abstract

The fact that perception and behavior depend on the simultaneous and coordinated activity of neural populations is well established. Understanding encoding through neuronal population activity is however complicated by the statistical dependencies between the activities of neurons, which can be present in terms of both their mean (signal correlations) and their response variability (noise correlations). Here, we review the state of knowledge regarding population coding and the influence of correlated variability in the electrosensory pathways of the weakly electric fish *Apteronotus leptorhynchus*. We summarize known population coding strategies at the peripheral level, which are largely unaffected by noise correlations. We then move on to the hindbrain, where existing data from the electrosensory lateral line lobe (ELL) shows the presence of noise correlations. We summarize the current knowledge regarding the mechanistic origins of noise correlations and known mechanisms of stimulus dependent correlation shaping in ELL. We finish by considering future directions for understanding population coding in the electrosensory pathways of weakly electric fish, highlighting the benefits of this model system for understanding the origins and impact of noise correlations on population coding.

## Interpretation of Population Activity Requires Considering Correlations Between the Activities of Different Neurons

Understanding the concerted activity of neural populations remains a central problem in systems neuroscience. While simultaneous recordings of multiple neurons (i.e., population activity) has become increasingly feasible across animal models and brain areas, interpreting these data are often complicated. This is because neuronal activities are often not independent of one another, but rather show correlations. Such correlations have been found almost ubiquitously across species and brain areas (for review see Cohen and Kohn, 2011; Doiron et al., 2016), and it has been shown that they can have substantial impact, of either detrimental or beneficial nature, on signal encoding performance at the population level (Averbeck et al., 2006; Salinas and Sejnowski, 2011). Albeit their acknowledged importance (for review see Nirenberg and Latham, 2003; Averbeck et al., 2006; Cohen and Kohn, 2011; Salinas and Sejnowski, 2011; Rothschild and Mizrahi, 2015; Kohn et al., 2016),the origins and the functional implications of correlations for coding remain poorly understood in many cases.

Here we review the current state of knowledge regarding how electrosensory neural populations encode behaviorally relevant stimuli in wave-type weakly electric fish. This model system is well described in terms of its anatomy and single cell physiology and readily accessible for *in vivo* recordings. As such, this system benefits from unique advantages when, e.g., considering realistic decoding of population activity and how this leads to behavior as well as for understanding population coding in more naturalistic experimental paradigms (e.g., when recording from unrestrained animals during active exploration and sensing).

### Decomposing Correlations

**FIGURE 1:**
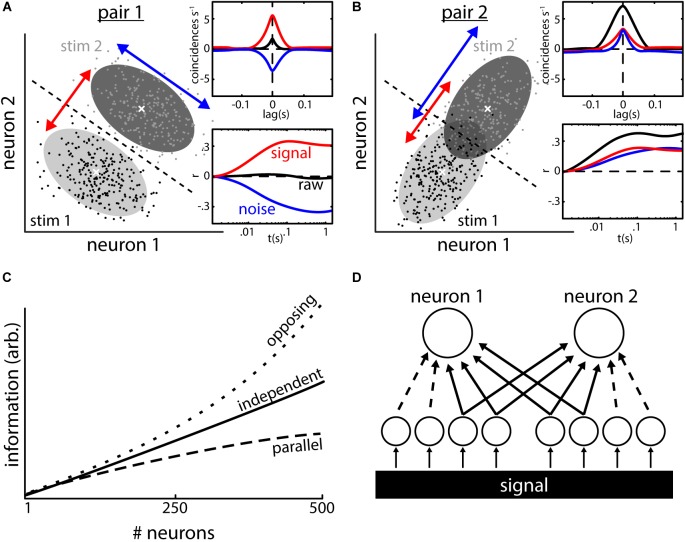
Types of neural correlations and their impact on population coding. **(A)** Responses (e.g., firing rate) of two neurons to repeated presentations of two different stimuli. The responses to stimulus 1 (black dots, light shading is 95% interval of the distribution) are lower than those to stimulus 2 (gray dots and dark shading) on average. The average responses (white crosses) co-vary positively (red arrow) indicating the presence of positive signal correlations. The trial-to-trial variabilities in the responses to repeated presentation of a given stimulus (e.g., scattering of black dots) co-vary negatively (blue arrow), which indicates the presence of negative noise correlations. In this example, the noise correlations aid stimulus discriminability compared to a case with independent responses (distributions would be circular). This is because noise correlations have an opposite sign compared to signal correlations, i.e., correlation structure is *opposite*, thereby leading to a decrease in the overlap between both distributions. Dotted line shows the best possible discrimination criterion, which allows for perfect discrimination in this case. Inset: quantification of the correlations shown in the example using cross-correlograms (CCGs, top) and spike count correlations as a function of timescale (bottom). **(B)** Same as **(A)** but with *parallel* correlation structure (i.e., signal and noise correlations are positive). Stimulus discriminability is impaired in this example: the overlap between distributions is increased due to the presence of noise correlations and using the discrimination criterion (dotted line) does not serve to discriminate between responses [compare to **(A)**]. **(C)** Even weak noise correlations have strong implications for coding on a population level. In absence of correlations, information in a population increases monotonically with increasing the number of neurons that are read out (solid line). With an opposite correlation structure [as in **(A)**], the amount of information surpasses the independent case very quickly (upper dotted line). With a parallel correlation structure [as in **(B)**], the growth of information is decreased and saturates. **(D)** Inputs to the two neurons consist of common (solid lines) as well as independent inputs (dotted lines). Signal correlations arise from inputs (independent AND shared) with similar tuning to a common signal. Noise correlations in turn arise from common inputs. Data in **(C)** illustrated after (Zohary et al., 1994; Averbeck et al., 2006).

When evaluating the statistical dependency between the spike trains of two neurons, their correlations (raw-correlation) can be decomposed into two types of correlations (Perkel et al., 1967). First, signal correlations, which are correlations between the mean activity of two neurons responding to stimuli. Second, noise correlations, which are correlations between the trial-to-trial variabilities of the neural responses to repeated presentations of a given stimulus (we will use the terms “noise correlations” and “correlated variability” as synonyms throughout this manuscript). It is important to note that simultaneous recordings are required to infer noise but not signal correlations. Theory predicts that the correlation structure (i.e., the relationship between signal and noise correlations) will determine their impact on information transmission (Averbeck et al., 2006; Kohn et al., 2016). To exemplify this, let us consider the simultaneous responses of a pair of neurons (Figure 1) to repeated presentations of two stimuli (black and light gray dots in Figures 1A,B). For each of the stimuli, the responses of the two neurons show variability and scatter around their mean (gray areas show the 95% probability distributions, white crosses indicate the means).

The neuron pair shown in Figure 1A has an opposing correlation structure: the average responses of the two neurons are positively correlated (i.e., both increase their mean response when stimulus 2 is presented vs. stimulus 1) and as such their signal correlations are positive (see insets, red curves). If the variabilities of the two neuron responses to repeated presentations of the same stimulus were independent, the probability distributions around the means would be circular in shape. Instead, they have an elliptical shape with the main axis being oriented from top left to bottom right. As such, the response variabilities are not independent but rather correlated. Indeed, whenever the response of neuron 1 is higher than its mean response, the response of neuron 2 tends to be lower than its mean response and vice versa. Thus the variabilities are negatively correlated, and noise correlations are negative (see insets, blue curves). This opposing correlation structure (positive signal and negative noise correlations, see orientation of red and blue arrows) is beneficial for stimulus encoding and by using a decision criterion (dotted line) it is possible to perfectly discriminate between population responses to the two different stimuli.

In Figure 1B, we show a different pair of neurons with a parallel correlation structure (i.e., positive signal and positive noise correlations). In this case, the distributions of the neural variabilities have an elliptical shape whose main axis is oriented from bottom left to upper right, which is parallel to the changes seen in mean responses to the different stimuli (white crosses, see orientation of red and blue arrows). As a result, the two response distributions show considerable overlap and the decision criterion (dotted line) results in impaired performance compared to the opposing correlation structure discussed above. This exemplifies that the presence of noise correlations can either be detrimental or beneficial for stimulus encoding and that their effect needs to be evaluated on a case by case basis.

The insets in Figures 1A,B show examples of how correlations are typically quantified. Cross correlograms (CCGs, inset top) quantify the number of coincident events per unit time relative to chance as a function of lag (i.e., the amount by which a spike train is shifted relative to the other). The integration and normalization of such CCGs produces a correlation coefficient that quantifies the correlation at infinite timescale (Shadlen and Newsome, 1998). Recently, however, the use of spike count correlations (inset bottom) has become more and more common. For this, the spike trains are binned into time windows of a defined width “t,” and the number of spikes falling within each bin is counted. The resultant spike count timeseries are compared by calculating the Pearson’s correlation coefficient. By reiterating the analysis with different width of the spike count window “t,” correlations can be analyzed at different timescales. Signal and noise correlations can be obtained from spike trains using standard computational methodology such as shuffle predictor and computing response residuals (Perkel et al., 1967).

In the absence of stimulation, raw-correlations between neural activities are often termed “baseline correlations.” These baseline correlations represent the limit that noise correlations will tend toward as stimulus amplitude goes to zero (Hofmann and Chacron, 2017). Therefore, it is expected that the presence of baseline correlations predicts the presence of noise correlation under stimulation.

### Effects of Correlated Variability on Stimulus Encoding

One could argue that the detrimental effects of noise correlations toward stimulus discrimination in the example shown (Figure 1B) might seem minimal. This is because most of the responses will still be categorized correctly based on the decision criterion (Figure 1B) and only a minor part of the responses will be attributed to the wrong stimulus. It should, however, be noted that the shown example considers only two neurons and that perception is typically determined by integrating the activities of much larger neural populations. It was shown theoretically that small pairwise correlations can have strong effects on signal encoding when large neural populations are considered (Zohary et al., 1994; Latham and Nirenberg, 2005; Schneidman et al., 2006) (Figure 1C). In the absence of noise correlations (i.e., when trial-to-trial variabilities are independent), the amount of information represented by the population activity grows as more neurons are considered for analysis (Figure 1C, solid line). The effects of trial-to-trial variability on the population information highly depend on the correlation structure. With an opposing structure (top dotted line; noise and signal correlations have an opposing sign), the growth of information quickly surpasses the independent case. In contrast, a parallel correlation structure (bottom dotted line; noise and signal correlations have the same sign) will lead to a reduction in information growth and quick saturation (Zohary et al., 1994). Thus, the presence of noise correlations alone does not suffice to assess their impact on signal encoding as they could be either detrimental (Zohary et al., 1994; Moreno-Bote et al., 2014) or beneficial (Abbott and Dayan, 1999; Romo et al., 2003) and their actual effect highly depends on the correlation structure as well as on the subsequent decoding by downstream brain areas. In that regard, various different decoders can be used (Pouget et al., 2000) and many studies have assumed linear decoders (i.e., relevant quantities are estimated based on weighted linear sums of neuronal responses) (Seung and Sompolinsky, 1993; Zohary et al., 1994; Sanger, 1996; Abbott and Dayan, 1999; Liu et al., 2013; Pitkow et al., 2015). Such decoders are attractive because they are easy to implement and optimize on neural data to quantify the effects of correlations. However, knowledge gained from these require comparison to physiologically plausible decoding strategies which are, in general, nonlinear and thus can in theory extract much more information than linear decoding strategies (Shamir and Sompolinsky, 2004). The effects of various decoders on determining how correlations influence information transmission has been reviewed in detail elsewhere (Kohn et al., 2016).

Recent studies have shown that noise correlations are not static but can change in magnitude based on various factors such as the animal’s state (Poulet and Petersen, 2008; Ecker et al., 2014; Erisken et al., 2014; Vinck et al., 2015), the animal’s attention (Steinmetz et al., 2000; Cohen and Maunsell, 2009), adaptation to stimuli (Gutnisky and Dragoi, 2008), or in a stimulus-dependent fashion (Chacron and Bastian, 2008; Snyder et al., 2014; Tan et al., 2014; Franke et al., 2016; Zylberberg et al., 2016). The plasticity of noise correlations greatly complicates understanding their effect on information coding.

### The Mechanistic Origins of Correlations *in vivo* Remain Poorly Understood

While correlations, both signal and noise, are found ubiquitously in the CNS, in many cases understanding their mechanistic origins remains elusive. On the one hand, it is clear that signal and noise correlations in pairs of neurons will highly depend on their input connectivity (Figure 1D). Signal correlations arise when two neurons receive inputs that encode the same signal and are also tuned to this signal (i.e., they both respond to this signal). Noise correlations are generally thought to arise because of shared neuronal input (Shadlen and Newsome, 1998; Renart et al., 2010; Kanitscheider et al., 2015). The activity of these common inputs (Figure 1D, solid lines) will influence the membrane potential of the receiving neurons similarly and thus introduce noise correlations between their spiking activities. Independent inputs (dotted lines) will usually dilute that stochasticity, thereby decreasing noise correlations potentially. Thus, noise correlations are likely to depend on the network architecture and activity (Bujan et al., 2015; Doiron et al., 2016). For example, it was shown that the balance between excitatory and inhibitory inputs might be one key determinant of correlation magnitude (Renart et al., 2010; Litwin-Kumar et al., 2011).

There has been progress made toward understanding the mechanistic origins of neural correlations and their effects on coding, and it has become clear that such understanding will require detailed knowledge of the anatomical connections. Nonetheless, the connections of neural networks forming circuits in the brain and their inputs and outputs are often numerous, divers, and highly complicated. Toward this end, the electrosensory pathway benefits from a relatively simple and well-characterized anatomy, which should be advantageous for the investigation of the basic mechanisms underlying correlated variability.

## Electrosensory Stimuli: Electrolocation and Social Interaction in Apteronotus

**FIGURE 2:**
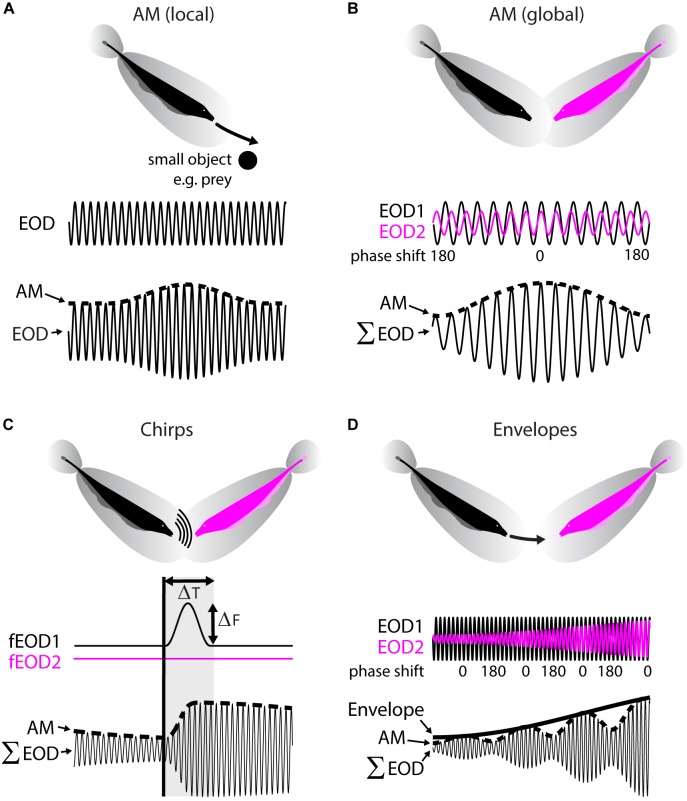
Natural electrosensory stimuli. **(A)** Local amplitude modulations (AM) of the EOD are caused by objects with a conductivity different to that of water (e.g., rocks or prey) during relative motion (top). The emitted EOD (middle) and the exact AM waveform will depend on the nature of the movement and the objects properties. The shown example (bottom), depicts an AM (dotted line) as it would be caused by the fish moving on a linear trajectory along a uniform conductive object (i.e., a metal sphere). Note that the EOD AM will be spatially localized and thus *not* spatially uniform across all receptors. **(B)** Global AMs are caused by interactions between the electric fields of conspecifics in proximity (top). The EODs emitted by each fish (middle, black, and magenta traces), will, due to their frequency difference, go in and out of phase repetitively (see phase shift). The periodic constructive and destructive interference between the signals will result in a compound signal (Σ EOD, bottom) with a sinusoidal AM at the frequency difference between the two EODs. This AM will be approximately spatially uniform across the fish’s skin. **(C)** Weakly electric fish communicate with conspecifics by emitting active modulations of their electric field called “chirps” (top). These consist of transient Gaussian-like increases in EOD frequency of one fish (middle) of a specific duration (ΔT) and frequency excursion amplitude (ΔF). As a result (bottom) the present AM (dotted line) is interrupted with a high-frequency transient (dotted line with gray shading). The resultant waveform of a chirp with a given ΔT and ΔF is very heterogeneous in *Apteronotus leptorhynchus* and depends on the beat phase at which the chirp is emitted. Thus, while the emitted signal of two chirps might be exactly the same, the waveform detected by the receiver will likely differ. **(D)** Relative motion between fish (top) will result in a change in EOD amplitude as seen from the focal fish (middle, see magenta trace, black fish is assumed as the focal fish). In the compound signal, (Σ EOD, bottom) the AM (dotted line) will, therefore, be amplitude modulated. Envelopes caused by relative motion typically have power at frequencies below 2 Hz and have been shown to be of behavioral relevance.

Wave-type weakly electric fish such as *A. leptorhynchus* emit a quasi-sinusoidal electric signal referred to as the “electric organ discharge” (EOD) thereby building up an oscillatory field surrounding their body (Figure 2A, top). It is important to realize that this ongoing EOD acts as a carrier signal during active sensing and that it is the perturbations of the EOD that carry information about the sensory environment. These are picked up by a distributed array of electroreceptors in the skin of the animal (Carr et al., 1982). The animal can detect both amplitude modulations (AMs) as well as frequency modulations (FMs) of the EOD via separate pathways. In the following, we will focus on the AM coding pathway and will henceforth refer to the AM as the stimulus.

Electrosensory stimuli occur in different behavioral contexts. During prey capture (Nelson and MacIver, 1999), animals detect and localize (i.e., “electrolocation”) small prey items that cause weak and spatially localized AMs of the EOD (Figure 2A). Several studies have shown that the resulting pattern of stimulation carries important information about the distance, size, and conductivity of an object and the relative speed and angle of the motion between object and fish (Rasnow, 1996; Nelson and MacIver, 1999; Nelson et al., 2002; Babineau et al., 2006; Hofmann et al., 2017; Pedraja et al., 2018).

Another behavioral context is that of interactions with conspecific fish (i.e., “electrocommunication”) (Ramcharitar et al., 2005; Kelly et al., 2008; Henninger et al., 2018). When two individuals are in close vicinity to one another, interaction between their EODs will create a sinusoidal stimulus (i.e., a beat) whose frequency is equal to the EOD frequency difference and ranges between a few Hz to several hundred Hz (Figure 2B). It is important to note that such stimuli are spatially diffuse and extend to most if not all the electroreceptors.

During social interaction, fish can emit short-term alterations of their EOD frequency with the purpose of active social communication (Hagedorn and Heiligenberg, 1985; Zupanc and Maler, 1993; Zakon et al., 2002; Benda et al., 2005; Zupanc et al., 2006; Hupé et al., 2008). Such events are called chirps and always occur on top of the beat (Figure 2C). There are different types of chirps (Zakon et al., 2002) and in the following we will focus on so called “small chirps,” which are typically aggressive call signals. Chirps are produced through brief (ΔT: 13–16 ms) and small (ΔF: 30–50 Hz) excursions in the EOD frequency of one fish (Figure 2C, middle). As a result, the periodic signature of the AM is interrupted by a high-frequency transient that resets the phase of the AM (Figure 2C, bottom). Importantly, for a given chirp with a fixed ΔF and ΔT, the exact waveform of the chirp will look very different depending on the AM phase at which the chirp is emitted. Chirps will cause diverse responses in pyramidal cells (for review see Marsat et al., 2012), but different chirp waveforms of the same chirp will give rise to similar behavioral responses (Hupé et al., 2008; Metzen et al., 2016a).

So far, we have only considered stimuli that consist of changes in the mean EOD amplitude. These are sometimes referred to as “first-order” stimuli. However, it is clear that rather than being stationary, fish move extensively during social interactions, thereby causing changes in the amplitude of the beat stimuli (Hupé and Lewis, 2008). Such “second-order” stimuli have been termed “movement envelopes” (Yu et al., 2012; Stamper et al., 2013; Metzen and Chacron, 2014). As an example, let us consider one fish looming toward a conspecific (Figure 2D, top). As seen from the perspective of the moving fish, the EOD amplitude of its conspecific will grow during the looming motion, thereby causing an increase in the beat amplitude termed envelope (Figure 2D, middle and bottom). Such movement envelopes typically contain power at very low (<1 Hz) frequencies (Fotowat et al., 2013; Metzen and Chacron, 2014). Movement envelopes will elicit behavioral responses in which the animal’s EOD frequency “tracks” the envelope stimulus (Metzen and Chacron, 2014).

Most studies investigating neuronal coding in weakly electric fish were performed in immobilized animals (note that the EOD persists after immobilization in species such as *A. leptorhynchus*). However, electrosensory behaviors consist of changes in the animal’s electric field and, as such, can be also be elicited in immobilized animals (Hitschfeld et al., 2009). Investigators have taken advantage of this fact to gain better understanding as to the nature of the electrosensory neural circuits that give rise to behavior (Heiligenberg, 1991). Recent studies have shown that the responses of electrosensory neurons to stimuli associated with different contexts strongly determine behavioral output (Deemyad et al., 2013; Huang et al., 2016, 2018; Metzen et al., 2016a, 2018; Metzen and Chacron, 2017). Some of these results pertaining to population coding are described below.

## The Anatomy of the Electrosensory Pathway

At the peripheral level, electrosensory stimuli (AM of the EOD) are detected by about 16,000 tuberous electroreceptors or electrosensory afferents (EA) that are distributed across the animals’ body and embedded in its skin (Carr et al., 1982). At baseline (i.e., in the absence of stimulation), each EA fires with a specific firing probability but phase locked to the EOD carrier wave and stimulation will cause changes in the firing probability (Scheich et al., 1973; Bastian, 1981).

**FIGURE 3:**
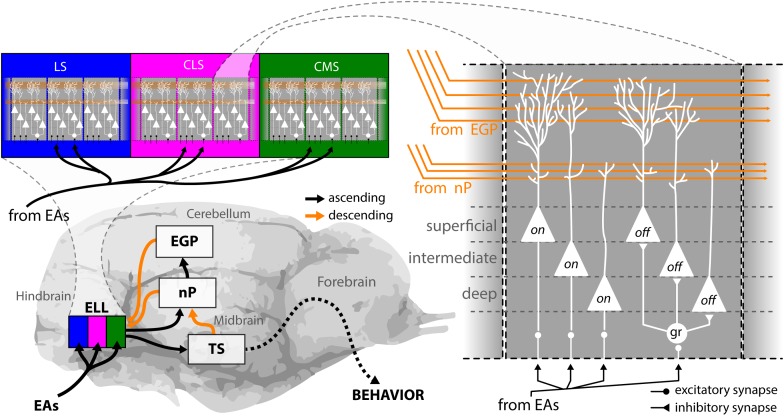
Anatomy of the electrosensory pathway. EOD AMs are detected by electroreceptors distributed in the fish’s skin, from where they send EAs to the electrosensory lateral line lobe (ELL) in the hindbrain (bottom left). The ELL is a cerebellum like structure with ascending (black arrows) and descending (orange arrows) projections and is organized in three parallel segments, the lateral (blue), the centro-lateral (magenta), and the centro-medial (green) segments (top left). The body surface is represented somatotopically in each segment. Moreover, pyramidal cells within all segments are arranged in a columnar organization with every column consisting of six cells (right). Three of these are on-type, as they receive direct excitatory input from EAs through basal dendrites therefore responding with an increase in firing rate to increases in EOD amplitude. The other three cells are Off-type, as they receive afferent signals via inhibitory interneurons (gr) and thus respond with a decrease in firing rate to increases in EOD amplitude. For each neuron type (i.e., On or Off), there is one superficial, one intermediate and one deep cell to be found within every ELL column. These differ in the amount of descending inputs they receive. Pyramidal neurons are the output neurons of the ELL that project to the midbrain torus semicircularis from where signals are processed and relayed to higher order brain areas to ultimately generate behavioral output. All pyramidal neurons receive descending inputs that originate from midbrain projections to the nucleus praeminentialis (nP) and projects in a somatotopically ordered fashion to the ELL (direct pathway). In addition, superficial and intermediate pyramidal neurons receive indirect descending inputs from the eminentia granularis posterior (EGP) onto their large apical dendritic arborizations in a spatially diffuse manner (indirect pathway). These inputs originate from the outputs of deep pyramidal neurons to EGP indirectly through nP. Descending inputs to ELL are excitatory via direct synapses between parallel fibers and apical dendrites and inhibitory through local interneurons in the molecular layer (not shown). EAs, electrosensory afferents; CLS, centrolateral segment; CMS, centromedial segment; EGP, eminentia granularis posterior; ELL, electrosensory lateral line lobe; gr, granule cell; LS, lateral segment; nP, nucleus praeminentialis.

EAs project to the hindbrain (Figure 3, bottom left), the first processing station in the brain, where they trifurcate to make synaptic contact with pyramidal cells in the electrosensory lateral line lobe (ELL). The ELL is organized in three parallel somatotopic maps of the body surface: the lateral, the centro-lateral, and the centro-medial segment (LS, CLS, and CMS) (Bell and Maler, 2005; Krahe et al., 2008; Krahe and Maler, 2014). All three segments are composed of columns as a repetitive motif (Figure 3, top left), with each column consisting of six different pyramidal neurons (Maler, 2009a) (Figure 3, right). Three of these neurons (“on”) receive direct excitatory input from EAs and respond with increases in their firing rate to increases in the AM. The three other neurons (“off”) receive the EA input via an inhibitory granular interneuron (“gr”) and thus instead respond to increases in the AM with decreases in firing rate (Maler et al., 1981; Saunders and Bastian, 1984; Bastian and Courtright, 1991). Pyramidal neurons are the sole output neurons of the hindbrain and project to the midbrain torus semicircularis (Figure 3, bottom left) where sensory information is further processed, and forwarded to various stages in the forebrain ultimately giving rise to behavior. There are three classes of pyramidal neurons: superficial, intermediate, and deep neurons, named after where their cell body is located within the pyramidal cell layer of the ELL (Maler et al., 1981). The three classes differ in terms of their cell morphology, physiology, and connectivity. Superficial cells have low baseline firing rates, large apical dendritic arborization that extend widely through the molecular layer, and receive huge amounts of descending inputs (orange), deep cells have high baseline firing rates and receive little to no descending inputs (Bastian, 1986; Bastian and Courtright, 1991; Bastian and Nguyenkim, 2001; Bastian et al., 2004; Chacron et al., 2005a). The properties of intermediate cells are in between the others.

There are two major types of descending pathways to ELL that originate from higher brain areas (Figure 3, orange). Inputs from the nucleus praeminentialis (nP, commonly termed direct feedback pathway) as well as from the eminentia granularis posterior (EGP, commonly termed indirect feedback pathway) form parallel fibers in ELL that make contact with the apical dendrites of pyramidal neurons. Direct contacts of parallel fibers with pyramidal neurons are excitatory, indirect contacts through interneurons in the molecular layer are inhibitory. Both types of descending inputs can strongly affect the responses of single ELL pyramidal cells to stimulation (Bastian, 1986; Bastian et al., 2004; Chacron et al., 2005c; Bol et al., 2011; Clarke and Maler, 2017; Metzen et al., 2018). The detailed anatomy and function of these pathways have been reviewed elsewhere (Bastian, 1999; Berman and Maler, 1999).

## Population Coding by Electrosensory Afferents

### Correlation-Based Coding of Chirps by Electrosensory Afferents

**FIGURE 4:**
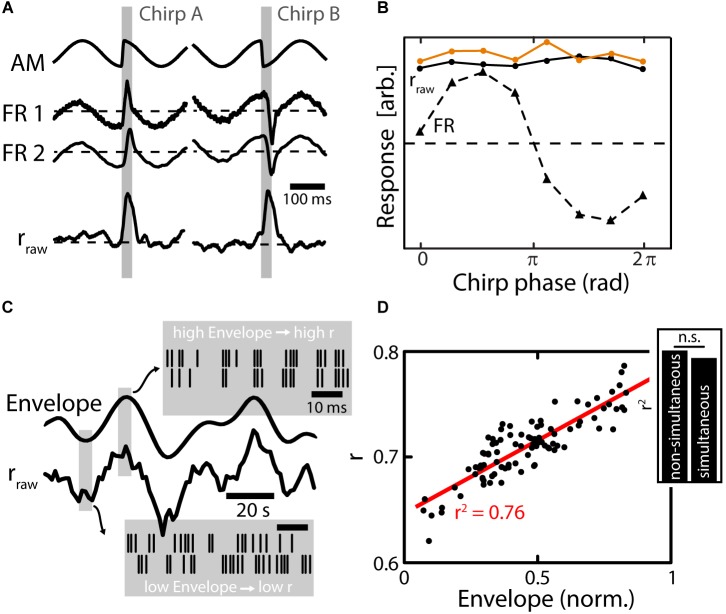
Population coding of electrosensory afferents. **(A)** Top: Stimulus waveforms (gray shading) resulting from chirps with a fixed ΔT and ΔF emitted during different phases of the AM (EOD not shown). The waveform of the chirp on the left consists of a sharp increase of the AM, while it is a sharp decrease for the chirp on the right. The social meaning of these chirps, however, is exactly the same as established in behavioral experiments. Middle: Firing rate responses of simultaneously recorded EAs. EAs encode the waveform of the AM faithfully, which results in very heterogeneous response waveforms between the two different chirps (compare left and right). Bottom: The time varying correlations between EAs increase during the chirp event. The increase in the correlation coefficient is similar between different chirps (left vs right). **(B)** Quantification of responses to chirps of different phases. Responses are very heterogeneous for the different chirp phases based on firing rate (FR, triangles and dotted line) while responses are invariant based on correlations (*r*_raw_ dots and solid black line). Importantly, behavioral responses were also invariant (orange dots and solid line) implying that correlations can better predict behavior than the single neuron firing rate. **(C)** Envelope signal (solid line on top) and time varying correlation coefficient (*r*_raw_) of two EAs. During low envelope amplitudes, the firing of EAs is heterogeneous (see raster plots in lower gray window) while during high envelope amplitudes firing is more similar between EAs (see upper gray window). The changes in correlation coefficients closely track the envelope signal (compare two solid black traces). **(D)** The relationship of correlation coefficients and envelope amplitude is linear and strong (for the shown example *r*^2^ = 0.76). Inset: similar results were obtained when using simultaneous and non-simultaneous recordings of EAs suggesting that noise correlations are of little relevance for signal encoding at the stage of afferents. Data in **(A,B)** from (Metzen et al., 2016a), in **(C,D)** from (Metzen et al., 2015b).

As described above, chirp waveforms of a given chirp are very heterogeneous in nature depending on the phase of the AM at which they are emitted (“chirp phase”). Nonetheless, chirps occur with equal probability at any phase of the beat during electrocommunication (Aumentado-Armstrong et al., 2015). The chirp waveforms can, depending on the chirp phase, consist of sharp increases, sharp decreases (Figure 4A, top traces, gray shading) or biphasic high-frequency transients in the AM. All these different chirp waveforms, however, were shown to elicit similar behavioral responses (Metzen et al., 2016a) suggesting that the social content of a given chirp is independent of the beat phase. At the level of EAs, the heterogeneous chirp waveforms will elicit very heterogeneous responses in the firing activities of single EAs (Figure 4A, middle curves) that have been well-characterized (Benda et al., 2005, 2006; Walz et al., 2014). As a result, the responses of EAs, in terms of their firing rate, are highly different depending on the chirp phase (Figure 4B, dotted line) while the behavioral responses are ultimately not (Figure 4B, orange line). Chirp stimulation increased the similarity of the firing patterns of pairs of EAs, thereby causing an increase in their correlations (Figure 4A, bottom) (Metzen et al., 2016a). These increases in correlations were very similar across the different possible waveforms of a given chirp (i.e., correlation-based responses were invariant) (Figure 4B, solid black line). This correlation response closely resembled the behavioral invariance to different chirp waveforms (Figure 4B, compare black and orange solid lines).

Interestingly, these results were similar for both simultaneous and non-simultaneous recordings. As explained above, non-simultaneous recordings cannot be used to infer noise correlations but only signal correlations. This suggests that changes in EA’s correlations are primarily driven by changes in signal correlations and that noise correlations between EAs are either negligible or do not affect such coding. This is supported by other studies showing that correlations between EA baseline activities are negligible except at the EOD frequency and higher harmonics (Chacron et al., 2005b). Based on recordings from several other processing stations (i.e., ELL and Torus semicircularis), Metzen et al. (2016a) show how EA correlations can be decoded in a physiologically plausible manner by combining and integrating parallel inputs along the ascending electrosensory pathway. Interestingly the correlation-based detectability of chirps depends on stimulus background: increasing beat frequency impairs detectability. Importantly the behavioral detection performance declines in parallel with correlation based coding performance (Metzen and Chacron, 2017). These results strongly suggest that correlations in EA activity are decoded by downstream brain areas to give rise to behavior.

### Correlation-Based Coding of Envelopes by Electrosensory Afferents

Recent studies have focused on understanding how EA’s respond to envelopes through changes in firing rate (Metzen and Chacron, 2015). While envelopes do not elicit changes of the EA’s average firing rate (as compared to baseline), it was found that they caused changes in the similarity of firing patterns (Figure 4C, gray boxes) and thus in the correlation between EA’s. Therefore, they could be detected when analyzing the firing patterns of the EA population. In fact the correlation magnitude in the EA population nicely tracks the envelope waveform (Figure 4C, solid lines) (Metzen et al., 2015b), and there was a strong relationship between the envelope and correlation (Figure 4D). This indicates that also second-order stimulus features are encoded through neuronal correlations, which might give rise to previously observed behavioral responses by which the animal’s EOD frequency tracks the detailed time course of the envelope in an almost one-to-one fashion (Metzen and Chacron, 2014).

As found during EA correlation encoding of chirps, results were almost independent of whether simultaneous or non-simultaneous recordings were used for the analysis (Figure 4D, inset), suggesting that changes in correlation are primarily, if not exclusively, driven by changes in signal correlations between EA’s. Interestingly, theory predicts that such envelope coding by correlated activity was optimal for a given level of variability (Metzen et al., 2015a; Grewe et al., 2017), a prediction that was verified experimentally (Metzen et al., 2015b). Moreover, such coding appears to be a general feature of sensory processing, with similar results found in the coding vestibular afferents (Metzen et al., 2015b) and acoustic processing (deCharms and Merzenich, 1996) in non-human primates, as well as in LGN of the cats visual system (Dan et al., 1998).

## ELL Pyramidal Neurons Exhibit Correlated Variability: Mechanisms and Implications for Coding

ELL pyramidal neurons receive convergent input from EA projections: Anatomical studies have shown that up to about 65% of inputs are shared between pyramidal neurons in neighboring columns (Maler, 2009b). As such, it is expected that, unlike EA’s, pyramidal cells in the hindbrain will display both signal and noise correlations, i.e., exhibit correlated variability. In this section, we will start by reviewing how correlations between the baseline activities of ELL pyramidal cells arise and demonstrate that these serve as a good predictor of noise correlations under stimulation. We then move on and review the state of knowledge regarding the presence and plasticity of noise correlations in ELL.

### Mechanisms Mediating Baseline Correlations

**FIGURE 5:**
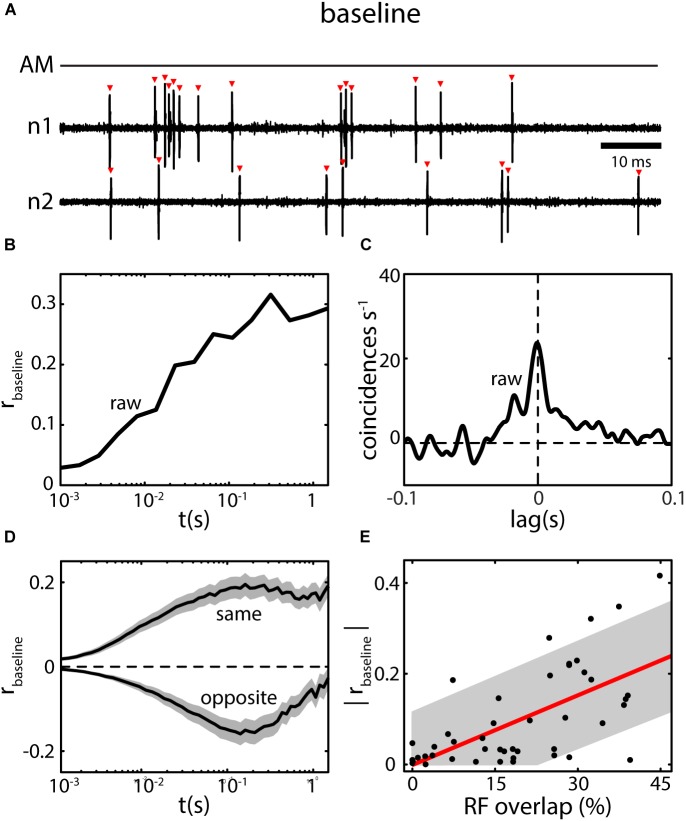
Baseline correlations in ELL pyramidal neurons. **(A)** ELL spiking activity of simultaneously recorded neighboring ELL neurons (n1 and n2) are typically not independent under baseline conditions (AM; no stimulus present). Many of the detected spikes (red triangles) in one neuron are nearly coincident with spikes in the other neuron. **(B)** Spike count correlations (*r*_baseline_; i.e., *r*_raw_ computed for spike trains recorded in absence of stimulation) as a function of time scale (*t*) for the neurons shown in **(A)**. Correlations are positive in the example and increase from low to high time windows. **(C)** The CCG for the example in **(A)** shows a broad peak of coincident events near lag 0, but also for higher lags (up to ca. 50 ms) coincident events are above chance level (0). By integrating and normalizing the CCG, a correlation coefficient can be obtained quantifying the correlations at all (i.e., infinite) timescales (for the example: *r* = 0.42). **(D)** Baseline correlations for pairs of pyramidal neurons consisting of same type neurons (i.e., On–On or Off–Off) are positive on average, for opposite type neurons (On–Off) negative on average (shown are mean ± SEM). Despite their sign, the overall magnitude is the same. **(E)** The magnitude of baseline correlations is closely related to the amount of receptive field overlap for the ELL CLS segment. Data in **(A–D)** from (Hofmann and Chacron, 2017), data in **(E)** from (Chacron and Bastian, 2008).

Under baseline conditions, ELL pyramidal neurons fire action potentials in an irregular pattern switching between bursts and single spikes (Figure 5A) (Bastian and Nguyenkim, 2001; Metzen et al., 2016b) with discharge rates in the range of a few to about 40 Hz (Bastian and Courtright, 1991). The spike trains of simultaneously recorded neighboring pyramidal neurons are typically correlated in the absence of stimulation (Chacron and Bastian, 2008; Hofmann and Chacron, 2017). The magnitude of these baseline correlations (i.e., raw-correlations recorded in absence of stimulation; see section “Decomposing Correlations” ), will typically increase from small (<10 ms) to large (>1 s) time windows (Figure 5B). Similarly, when calculating a cross-correlogram (CCG) a prominent peak near lag zero is visible, but coincident events above chance level are also found at higher lags, e.g., at 50 ms or higher (Figure 5C). The magnitude of baseline correlations is independent of the difference in firing rate between the neurons in a pair and is stationary over time (Hofmann and Chacron, 2017). Baseline correlations are on average positive between pairs of the same type (i.e., on–on and off–off) and negative between opposite type pairs (on–off) (Figure 5D). By mapping the receptive fields (RF, the area on the skin or in the environment within which a stimulus causes a response in the neuron) of pyramidal neurons, Chacron and Bastian (2008) were able to show a positive correlation between the amount of RF overlap of neuron pairs and their baseline correlation magnitude (Figure 5E). Such RF overlap is likely to be caused by shared EA input between pyramidal neurons (Maler, 2009a).

**FIGURE 6:**
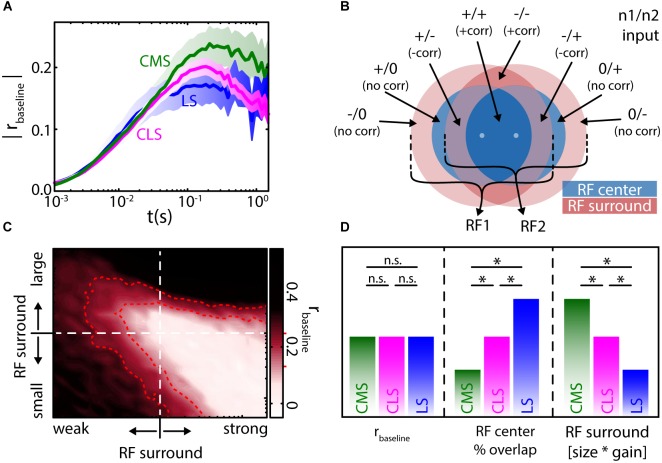
Baseline correlation magnitudes are similar across the ELL segments and determined by receptive field organization. **(A)** Population averages of baseline correlation magnitude as a function of timescale for the three ELL segments (see labels, shown are mean ± SEM). Despite varying degrees of receptive field (RF) overlap, the average magnitude of correlations was similar in all segments. **(B)** Two adjacent RFs (RF1 and RF2, respectively) with a center-surround (blue–red) organization will have up to eight areas over RF overlap. The inputs from these areas will be correlated (+*corr*), negatively correlated *(–corr*) or uncorrelated (*no corr*). The relation of these areas (in terms of size and strength) will determine the correlations between the inputs that the two pyramidal neurons receive. **(C)** In a numerical simulation, varying the RF structure (i.e., relative size and strength of the RF surround) led to differences in the magnitude of baseline correlations (all values at *t* = 100 ms). Interestingly, physiologically plausible magnitudes [*r* ≈ 0.2, compare to **(A)**] of correlations can be found for very different RF relations (area enclosed by red dotted lines, see red markers on colorbar). **(D)** From the numerical simulations and previously published qualitative physiological data (Shumway, 1989), the similar magnitudes in correlations between the segments (left) can, despite the varying degree of RF center overlap (middle), be explained by a compensation through the RF surround that varies (antagonistically to RF center overlap) between the segments (right). All data from (Hofmann and Chacron, 2017). ^∗^Statistical significance; n.s., not significant.

The average RF size and overlap between neighboring neurons was estimated to differ between ELL segments and decrease from the lateral segment (LS) over the centro lateral segment (CLS) to the centro medial segment (CMS) (Maler, 2009a). Based on the relationship between baseline correlation magnitude and RF overlap (Figure 5E) one would consequently predict baseline correlations to decrease from LS to CMS. This is not true, however, as the correlation magnitudes were found to be similar on average, in all three segments (Figure 6A) (Hofmann and Chacron, 2017). These similar magnitudes of baseline correlations most likely originate from different RF properties as described below.

ELL RFs are organized in an antagonistic center-surround organization (Shumway, 1989; Bastian et al., 2002). This means, for an on-type cell, stimulation within the RF center will cause an increase in firing rate, while stimulation within the RF surround will instead cause a decrease in firing rate. Thus, when considering the RFs of two neighboring ELL pyramidal neurons, one must consider up to eight different areas of RF overlap, depending on their spacing (Figure 6B). These areas will give either excitatory (“+”) or inhibitory (“-”) input to each of the two neurons, or will not project to a given neuron (“0”). Comparing the inputs, each neuron receives from each of the areas of overlap, one can expect these signals to be positively correlated (“+corr”), negatively correlated (“-corr”), or not correlated (“no corr”) (Figure 6B, labels). The balance between these inputs will determine the amount of input correlations. The correlation magnitude measured in a pair of pyramidal neurons will depend on their input correlations and the amount of correlation transfer in these neurons (Shea-Brown et al., 2008; Bujan et al., 2015).

Using mathematical models and numerical simulations, it was found that changing the relative strength and size of the RF surround relative to that of the RF center impacts the magnitude of baseline correlations (Figure 6C). Interestingly, many and very different RF topographies and balances led to correlation magnitudes within the physiological range (Hofmann and Chacron, 2017) (Figure 6C, red). As such, it is not only interactions between the RF centers but also interactions between the centers and the surrounds, as well as interactions between the RF surrounds themselves that contributes toward determining correlation magnitude. Therefore, the similar correlation magnitudes seen across the three ELL segments occur because decreases in RF center overlap when going from LS to CMS are effectively “compensated for” by the concomitant impact of signals from the RF surrounds (Figure 6D) (Shumway, 1989; Hofmann and Chacron, 2017). As mentioned above, the presence of baseline correlations in the absence of stimulation strongly suggests that noise correlations will be present during stimulation.

### Baseline Correlations Predict Noise Correlations Under Stimulation in ELL Pyramidal Cells

**FIGURE 7:**
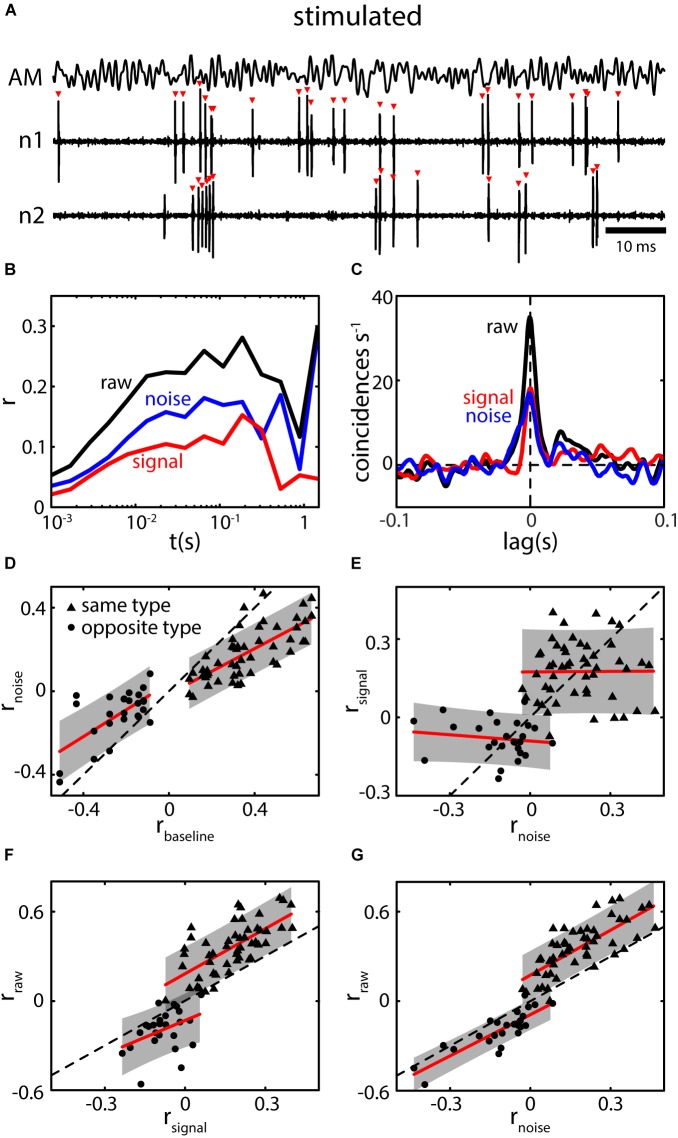
Baseline correlations predict the presence of noise correlations during stimulation. **(A)** Spiking activity of two neighboring pyramidal neurons (n1 and n2) during stimulation with a 0–120 Hz AM (AM, top trace). Spiking pattern encodes the AM while the average firing rate of neurons increased very little (shown neurons are the same as in Figures 5A–C). **(B)** Spike count correlations (*r*) as a function of time window (*t*) for raw (black), signal (red), and noise correlations (blue). Correlation structure of the shown example is parallel, i.e., both signal and noise correlations are positive. **(C)** CCGs for raw signal and noise correlations show prominent peaks near lag 0 extending to lags of ca. 20 ms. Note that, while the absolute peak of raw correlations is higher compared to baseline correlations (Figure 5C), the width of the peak is reduced. Correlations coefficient as determined from the CCG were: *r*_raw_ = 0.27 and *r*_noise_ = 0.12. **(D)** Relation between the magnitude of noise (*r*_noise_) and baseline correlations (*r*_baseline_) for same (triangles) and opposite (dots) type pairs. Note that, for both datasets a strong positive relation was found. While the sign is preserved the magnitude of *r*_noise_ is slightly reduced compared to *r*_baseline_ as the slope of the fits (red lines) are lower than that of the identity line (dotted line). The relationship shows that based on the presence of correlations under baseline conditions, noise correlations can be expected to be present under stimulation. The gap between the two population arises as only pairs with an absolute *r*_baseline_ above 0.1 were included in the analysis (see also Chacron and Bastian, 2008). **(E)** The magnitudes of signal and noise correlations were not systematically dependent on each other (red lines, fit to individual datasets, slopes were not significant). However, their sign seems to be preserved in general, i.e. correlation structure in ELL is on average parallel. **(F,G)** Raw correlations as a function of signal **(F)** and noise correlations **(G)**. In both cases, strong and significant relationships were found indicating that both components contribute to the overall correlation coefficient. However, noise correlations vary over a larger range, and the relationship was stronger suggesting that the impact of noise correlations slightly outweighs that of signal correlations. Data in **(A–C)** re-analyzed from Hofmann and Chacron (2017). Data in **(D–G)** reanalyzed from Chacron and Bastian (2008).

Under stimulation, pyramidal neurons will typically encode the stimulus waveform through changes in firing pattern while the overall firing rate changes only minimally on average (Figure 7A). This is generally attributed to gain control and the cancelation of redundant signals via descending pathways (i.e., indirect feedback) (Bastian, 1986, 1999; Bastian and Bratton, 1990; Bratton and Bastian, 1990; Chacron et al., 2005c) causing pyramidal neurons to adapt to both first- and second-order stimuli (Bastian et al., 2004; Huang et al., 2016; Zhang and Chacron, 2016). Simultaneously recorded spike trains of pyramidal neurons will display both signal and noise correlations (Figures 7B,C). Using previously published data (Chacron and Bastian, 2008; Hofmann and Chacron, 2017), we investigated the relationship between baseline correlations and noise correlations and found a strong relationship between them (Figure 7D). This confirms our earlier prediction that baseline correlations are a precursor for the presence of noise correlations under the assumption of weak stimulus amplitudes (Hofmann and Chacron, 2017). We note that the magnitude of noise correlations was systematically lower compared to the magnitude of baseline correlations (compare slope of fits to identity line) while the sign was preserved between same and opposite type pairs (Figure 7D, dots vs. triangles).

We further compared noise and signal correlations and found that the correlation structure in ELL is mostly parallel (i.e., signal and noise correlations typically have the same sign) (Figure 7E, compare also to Figures 1A,B). However, when considering either same or opposite-type pairs separately, there was no significant relationship between signal and noise correlation magnitudes (Figure 7E). As expected, both signal and noise correlations seem to contribute to the overall (raw-) correlation in ELL as, for both, significant relations were found (Figures 7F,G). The relationship between raw and noise correlations extends over a larger range (range of *r*_noise_: -0.35 to 0.39) as compared to the relation between raw and signal correlations (range of *r*_signal_: -0.28 to 0.37). Furthermore, the spread of the data is less (error areas of the fits are smaller). This could be seen as indication that noise correlations more strongly determine raw correlations between ELL spike trains than signal correlations are.

Based on the observed correlation structure (Figure 7E), one would predict that information transmission is compromised when assuming a decoder that relies on the linear sum of responses. It is, however, important to note that experimental data has shown that such a decoding scheme is most likely not completely accurate (Vonderschen and Chacron, 2011; Aumentado-Armstrong et al., 2015). Nevertheless, these results highlight the important fact that noise correlations between ELL pyramidal cell activities should not be assumed to be negligible and cannot be ignored when investigating population coding (Lewis and Maler, 2001; Marsat and Maler, 2010; Jung et al., 2016; Allen and Marsat, 2018).

### Plasticity of Correlated Variability in ELL

**FIGURE 8:**
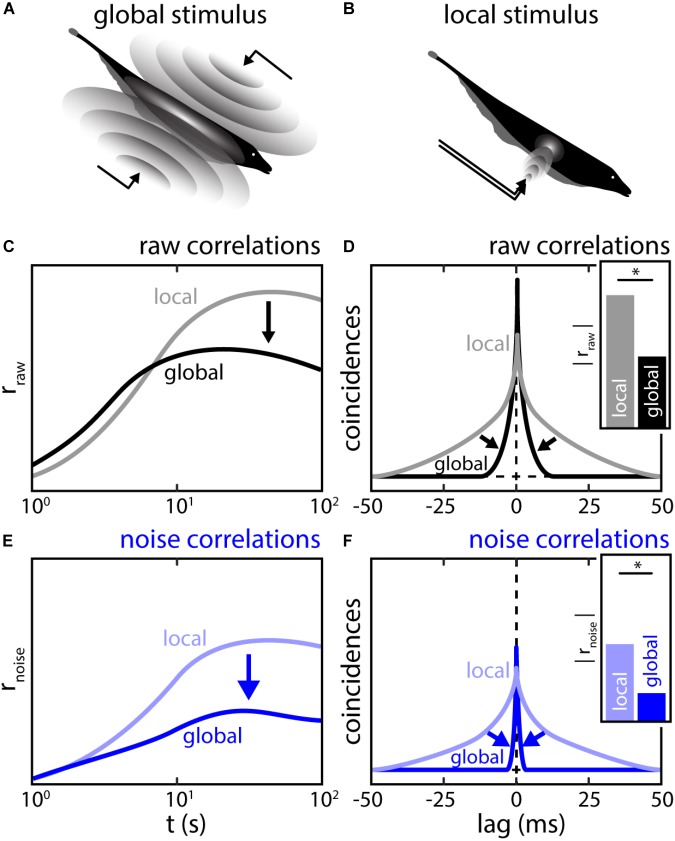
The spatial structure of stimulation shapes the magnitude and timescale of correlations between ELL pyramidal neuron activities. **(A)** Global stimulation resembles interaction between the electric field of conspecifics (compare Figures 2B,C). During such a stimulation, the sensory surface is stimulated uniformly with all EAs receiving a similar signal. At sufficient strength, global stimulation will activate descending inputs to ELL. **(B)** Local stimulation resembles electrolocation signals, i.e., interactions between the electric field and objects in the environment (compare Figure 2A). During such a stimulation, only a sub-portion of the sensory surface is stimulated. Under local stimulation, neuronal feedback is inactive **(C,D)** Population raw correlations during local (gray) and global (black) stimulation shown as spike count correlations as a function of timescale **(C)** and CCGs **(D)**. Correlations at high timescales are strongly reduced while correlations at low timescales may be slightly increased. Inset: the correlation coefficients as obtained from the CCGs were strongly reduced under global stimulation. **(E,F)** Same as **(C,D)** but for population noise correlations. Similar, to raw-correlations, a massive reduction of noise correlations was found during global stimulation. This implies that the reduction in noise correlations is driving the reduction in raw-correlations. ^∗^Statistical significance.

Similar to what is reported in other brain areas, noise correlations in ELL are highly plastic (see also “Effects of Correlated Variability on Stimulus Encoding” section). Indeed, it was shown that their magnitude strongly depend on the stimulus’ spatial extent (Chacron and Bastian, 2008; Litwin-Kumar et al., 2012; Simmonds and Chacron, 2015). Specifically, correlations in a given pair are low when using stimuli whose spatial extent mimics those caused by conspecifics (global stimulation; Figure 8A) compared to when using stimuli with the same temporal profile but whose spatial extent mimics those caused by prey (local stimulation; Figure 8B).

This effect is timescale specific: on short timescales (<10 ms) correlations slightly increased under global stimulation while on longer timescales (>10 ms), correlations strongly decreased (Litwin-Kumar et al., 2012) (Figures 8C,D, arrows). Litwin-Kumar et al. (2012) showed that this correlation shaping was in part due to changes in signal correlation, which reflects previously described changes in the response properties of single ELL pyramidal neurons (Chacron et al., 2005c; Chacron, 2006). Moreover, noise correlations were in general weaker under global stimulation than under local stimulation (Figures 8E,F, arrows).

**FIGURE 9:**
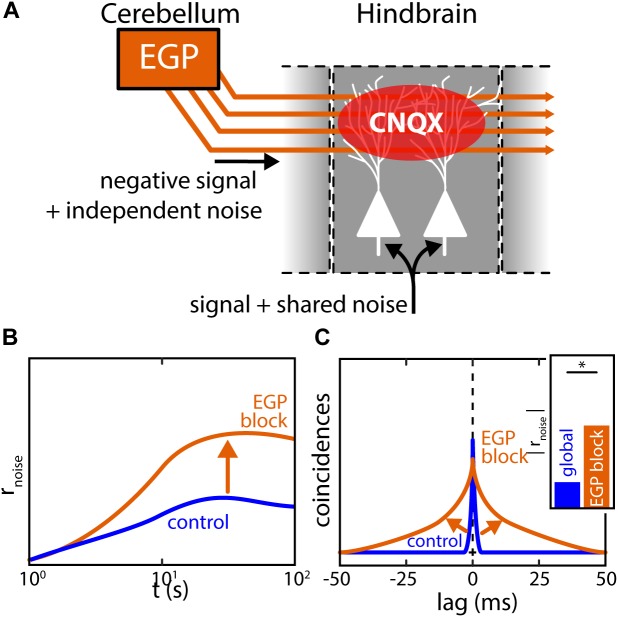
Pharmacological inactivation of descending inputs increases ELL correlations. **(A)** Feedforward projections of EAs to ELL pyramidal neurons are sources or shared noise generating noise correlations between neurons in close vicinity. Descending inputs from the EGP (indirect feedback) are spatially diffuse and carry independent noise. Feedback activation will thus dilute noise correlations as predicted by computational modeling leading to a decrease of noise correlation under global stimulation. The descending inputs from EGP can be pharmacologically blocked by releasing the agent CNQX in the vicinity of the apical dendrites interrupting synaptic transmission. **(B,C)** Population noise correlations during global stimulation and inactivation of the indirect EGP feedback through application of CNQX to the apical dendrites of ELL neurons. While noise correlations are low under global stimulation (*control*, blue), a large increase in noise correlations was found after feedback inactivation (*EGP block*, orange). In fact, noise correlations after feedback inactivation closely resembled those observed under local stimulation. Data illustrated after (Chacron and Bastian, 2008; Litwin-Kumar et al., 2012; Simmonds and Chacron, 2015). ^∗^Statistical significance.

It was shown, both by mathematical modeling and experimental manipulation, that the reduction of noise correlations under global stimulation is due to activation of the indirect feedback pathway (Simmonds and Chacron, 2015). This descending input pathway is diffuse and activated only under global but not local stimulation (Bastian et al., 2004). As explained above (see section “The Mechanistic Origins of Correlations *in vivo* Remain Poorly Understood”), noise correlations likely arise due to the shared noise in common inputs. For ELL, these are the afferent inputs from EAs (Figure 9A, bottom). As for the descending inputs, it is assumed that the granule cells within the EGP do not fire in the absence of stimulation but are active during stimulation. Further, it is assumed that the trial-to-trial variability in the granule cell firing activities contribute independent noise to the pyramidal cells due to the diffuse nature of the descending inputs (Figure 9A, left). Activation of the indirect feedback during global stimulation will therefore “dilute” noise correlations between pyramidal cells. Indeed, experimentally blocking this pathway during global stimulation led to an increase in noise correlations supporting this hypothesis (Figures 9B,C, arrows). As such, the descending inputs during global stimulation can be implicated as one functional component with which correlation plasticity is achieved in ELL. If and how this affects the processing of behavioral relevant signals is discussed below.

## Future Directions

In the following, we highlight interesting future avenues of research on population coding in the electrosensory system.

Noise correlations have been shown within the CLS segment (Chacron and Bastian, 2008; Simmonds and Chacron, 2015). Furthermore, the presence of baseline correlations was shown for all segments (Hofmann and Chacron, 2017) which, with the re-analyzed data presented here (Figure 7D), strongly implies that noise correlations will be present during stimulation in all ELL segments. Systematic assessments of differences in noise correlations between pairs of different pyramidal neuron types (i.e., superficial, intermediate, and deep) have not been done so far. Baseline correlation magnitude was reported to weakly correlate with the average baseline firing rate in CLS pairs (Hofmann and Chacron, 2017), suggesting that pairs of deep cells tend to display slightly higher correlation magnitude. Whether this holds true for noise correlations under stimulation has not been investigated to date. Further studies are also needed to assess how pyramidal cell heterogeneities affect correlation plasticity. Specifically, as deep cells receive less descending inputs in comparison to intermediate and superficial neurons, we predict that the reduction in noise correlation during global stimulation will be less pronounced in these cell pairs.

The initial estimation of correlation structure (Figure 7E) suggests that noise correlations might influence signal encoding in a detrimental fashion with respect to many of the analytical tools that were used in the past. However, it is important to note that it remains to be shown directly if and how they influence signal encoding, which will require evaluation from the decoding perspective also. For this, recordings from areas downstream of ELL such as the midbrain torus semicircularis will be required. There, the diversity of cell classes and response properties (Vonderschen and Chacron, 2011; McGillivray et al., 2012; Aumentado-Armstrong et al., 2015; Sproule et al., 2015) could imply that different decoding strategies are used for different stimuli.

One important area of research concerns how natural electrocommunication stimuli (i.e., chirps) are encoded within the electrosensory pathway. As mentioned above, most studies have focused on how single EA’s or ELL pyramidal cells encode such stimuli. While extrapolations to the population level were attempted, the potential effects of noise correlations were generally neglected (Marsat et al., 2009; Allen and Marsat, 2018). However, the fact that the baseline activities of LS pyramidal cells are correlated at timescales commensurate with those of chirps suggests that noise correlations need to be taken into account in future studies. Simultaneous recordings of ELL neurons during stimulation with chirps will be required to verify the above prediction and ascertain their effects on population coding. Further, the potential impact of noise correlations on population coding of chirps will require investigation of the decoding in downstream midbrain neurons. These integrate converging inputs from ELL pyramidal neurons and experimental studies have shown that some midbrain neurons, due to their non-linear integration of inputs from on- and off-type ELL pyramidal neurons, responded to chirps in a selective manner (Vonderschen and Chacron, 2011; Aumentado-Armstrong et al., 2015; Metzen et al., 2016a). Future studies should consider these more physiologically realistic decoding schemes, in particular we predict that pooling the activities of on- and off-type cells will reduce their overall responses to the beat, thereby making the response to the chirp more detectable.

With regard to electrosensory envelopes, previous studies have largely focused on understanding the encoding by single ELL pyramidal cells (Huang and Chacron, 2016; Huang et al., 2016; Zhang and Chacron, 2016; Metzen et al., 2018). Further studies are needed to understand the role and impact of noise correlations on population coding of envelopes. Here, responses of on- and off-type ELL pyramidal cells, while responding in andout of phase to first-order stimuli, respectively, actually respond largely in phase to second order stimuli such as envelopes (Huang and Chacron, 2016). From this, one would expect to find ELL signal correlations to be positive with regard to the envelope on average. However, the experimentally observed negative baseline correlations between opposite type pairs, would predict that these display negative noise correlations in response to envelopes. As such, noise correlations are predicted to be beneficial for envelope coding in such pairs. Further studies are needed to verify this prediction.

Finally, it should be noted that theoretical studies have suggested that noise correlations themselves could directly encode stimuli, therefore forming an independent channel of information transmission in the brain (Averbeck et al., 2006). The documented correlation plasticity in ELL, together with the nature of electrosensory stimuli being intertwined with an active sensing carrier signal, could be an example in which such a correlation code is realized in the brain. If plasticity of noise correlations can be found due to stimulus attributes other than the stimulus spatial extend, is unclear, however, and remains to be investigated.

While many of the above discussed assessments of neuronal encoding are based on recordings from immobilized animals, it is important to note that weakly electric fish, on top of being able to display electrical behaviors when immobilized, show elaborate behaviors and astonishing cognitive abilities and are getting more and more attention for the study of various aspects of active sensing behaviors (Nelson and MacIver, 2006; Engelmann et al., 2008; von der Emde et al., 2010; Hofmann et al., 2013, 2017; Pedraja et al., 2018). Recent technological advances such as electrophysiological recordings from freely moving aquatic animals are rapidly evolving (Fotowat et al., 2013; Vinepinsky et al., 2017). Being able to perform such recordings in freely behaving electric fish will allow to combine the investigations of population coding aspects in active sensing contexts – two of the most prominent research streams in neuroscience. Specific questions will likely be: How are active sensing movements generated and controlled through neuronal populations, how do population codes contribute to decision making during active sensing movements, and how do neuron populations encode sensory signals discriminating re- and ex-afferent signals at the population level. Based on the vast body of knowledge regarding behavior, anatomy and physiology weakly electric fish promise to evolve into an exciting model system to study the neuronal control of active sensing behaviors.

## Author Contributions

VH and MC conceived the study, reviewed and edited the text and figures. VH curated and re-analyzed the data, prepared the figures, and wrote the initial draft.

## Conflict of Interest Statement

The authors declare that the research was conducted in the absence of any commercial or financial relationships that could be construed as a potential conflict of interest.
